# Cardiovascular and Renal Comorbidities Included into Neural Networks Predict the Outcome in COVID-19 Patients Admitted to an Intensive Care Unit: Three-Center, Cross-Validation, Age- and Sex-Matched Study

**DOI:** 10.3390/jcdd10020039

**Published:** 2023-01-23

**Authors:** Evgeny Ovcharenko, Anton Kutikhin, Olga Gruzdeva, Anastasia Kuzmina, Tamara Slesareva, Elena Brusina, Svetlana Kudasheva, Tatiana Bondarenko, Svetlana Kuzmenko, Nikolay Osyaev, Natalia Ivannikova, Grigory Vavin, Vadim Moses, Viacheslav Danilov, Egor Komossky, Kirill Klyshnikov

**Affiliations:** 1 Department of Experimental Medicine, Research Institute for Complex Issues of Cardiovascular Diseases, 6 Sosnovy Boulevard, 650002 Kemerovo, Russia; 2 Department of Epidemiology, Kemerovo State Medical University, 22a Voroshilova Street, 650056 Kemerovo, Russia; 3 Kuzbass Regional Infectious Diseases Clinical Hospital, 43b Volgogradskaya Street, 650036 Kemerovo, Russia; 4 Kuzbass Regional Clinical Hospital, 22a Oktyabr’skiy Prospekt, 650061 Kemerovo, Russia; 5 Politecnico di Milano, 32 Piazza Leonardo da Vinci, 20133 Milan, Italy; 6 Faculty of Computer Science and Technology, Saint Petersburg Electrotechnical University, 5 Professora Popova Street, 197022 Saint Petersburg, Russia

**Keywords:** COVID-19, machine learning, neural networks, prognostication, coronary artery disease, chronic kidney disease, blood urea nitrogen, C-reactive protein, lymphocyte count, neutrophil-to-lymphocyte ratio

## Abstract

Here, we performed a multicenter, age- and sex-matched study to compare the efficiency of various machine learning algorithms in the prediction of COVID-19 fatal outcomes and to develop sensitive, specific, and robust artificial intelligence tools for the prompt triage of patients with severe COVID-19 in the intensive care unit setting. In a challenge against other established machine learning algorithms (decision trees, random forests, extra trees, neural networks, k-nearest neighbors, and gradient boosting: XGBoost, LightGBM, and CatBoost) and multivariate logistic regression as a reference, neural networks demonstrated the highest sensitivity, sufficient specificity, and excellent robustness. Further, neural networks based on coronary artery disease/chronic heart failure, stage 3–5 chronic kidney disease, blood urea nitrogen, and C-reactive protein as the predictors exceeded 90% sensitivity and 80% specificity, reaching AUROC of 0.866 at primary cross-validation and 0.849 at secondary cross-validation on virtual samples generated by the bootstrapping procedure. These results underscore the impact of cardiovascular and renal comorbidities in the context of thrombotic complications characteristic of severe COVID-19. As aforementioned predictors can be obtained from the case histories or are inexpensive to be measured at admission to the intensive care unit, we suggest this predictor composition is useful for the triage of critically ill COVID-19 patients.

## 1. Introduction

The COVID-19 pandemic has been a tremendous challenge for healthcare and society, being a confirmed cause of >636 million cases and >6.6 million deaths worldwide [1]. However, this healthcare burden led to the unprecedented accumulation of big clinical data, which have been processed and translated into artificial intelligence (AI) tools for the prognostication of COVID-19 [2,3,4]. Most of the machine learning (ML) studies (≈75%) focused on chest X-ray images and chest computed tomography data [5,6,7], demonstrating an area under the receiver operating characteristic curve (AUROC) of ≥0.9 in diagnostics and prognostication tasks. In contrast, complete blood count and routine biochemical markers have been less represented in ML approaches, though reaching similar AUROC values in combination with clinical risk factors such as comorbidities [8,9,10,11,12]. Moreover, an ensemble of laboratory markers has been suggested even for COVID-19 diagnosis [13,14,15,16], but the efficacy of this approach seems limited to pandemics, as having an unacceptably high risk of false positives related to other infectious diseases [17]. However, the rapid prognosis of COVID-19 using routine markers such as complete blood count or C-reactive protein might be a cost-efficient solution for developing and least-developed countries in case of pandemics caused by other SARS-CoV-2 mutants. Such a strategy conceives significant acceleration of the triage for selecting critical patients to commence the appropriate treatment immediately and for the optimizing of the workflow within the health facilities [18]. Accuracy of the predictions made by the corresponding ML tools fluctuates from 81 to 96%, including age, reduced oxygen saturation, increased serum lactate dehydrogenase, C-reactive protein, and impaired kidney function as major predictors of COVID-19-associated death [19,20,21,22,23,24,25,26,27]. Yet, these factors and their relative impact on mortality significantly vary between the countries and hospitals, probably due to distinct treatment protocols [19,20,21,22,23,24,25,26,27].

Another important issue that is often omitted in epidemiological and ML studies of COVID-19 is the close correlation of many predictors to age and sex, two inherent features of every patient. The investigations comparing the age- and sex-matched patients using ML approaches are currently limited to the deep profiling of circulating proteins (including cytokines), lipids, metabolites, and miRNA collected before or around the time of hospital admission and predicting the COVID-19 positivity [28,29,30,31] or disease severity [30,31,32,33,34,35], too small single-center studies of coagulation/fibrinolysis markers in predicting mortality [36], and to the studies of the lung computed tomography images [37] or electrocardiograms [38] to diagnose [37] or exclude [38] COVID-19. The matching of study cohorts by age and sex reduces the background behind the risk factors and clarifies the pathophysiological links between the comorbidities, organ damage, and serum biomarkers.

Strikingly, multicenter, age- and sex-matched ML studies aiming to develop AI tools for the accurate prediction of COVID-19-related mortality using non-specific predictors (i.e., comorbid conditions and complete blood count or routine biochemical parameters) have not been carried out hitherto. Moreover, evolving SARS-CoV-2 strains and increasing vaccination rates may also change the diagnostic and predictive value of different factors over time [18]. Here we conducted a three-center, cross-validation, age- and sex-matched study to design sensitive and specific ML tools for the prediction of the fatal outcome in patients admitted to the intensive care unit (ICU) because of severe COVID-19 using routinely available clinical and laboratory data. Out of all tested ML algorithms (decision trees, random forests, extra trees, neural networks, k-nearest neighbors, and gradient boosting: XGBoost, LightGBM, and CatBoost) and multivariate logistic regression as a reference, neural networks demonstrated the highest efficiency measured by percent of correct predictions (>90% sensitivity and >80% specificity) and AUROC (0.86) as well as the highest robustness to the heterogeneity of datasets across the study centers. Among the most valuable predictors included in the best neural networks was past/present medical history of coronary artery disease or chronic heart failure, stage 3–5 chronic kidney disease, blood urea nitrogen, and C-reactive protein. We suggest that our neural networks can be exploited for the triage of critically ill COVID-19 patients to adjust the treatment promptly, thereby improving the prognosis.

## 2. Materials and Methods

### 2.1. Patients

The study was conducted according to the latest revision of the Declaration of Helsinki (2013), and the donor study protocol was approved by the Local Ethical Committee of the Research Institute for Complex Issues of Cardiovascular Diseases (Kemerovo, Russia, protocol code 2020/07/1, date of approval: 9 July 2020), Local Ethical Committee of Kuzbass Regional Infectious Diseases Clinical Hospital (Kemerovo, Russia, protocol code AK-02, date of approval: 14 July 2020), and Kuzbass Regional Clinical Hospital (Kemerovo, Russia, protocol code OKB-633, date of approval: 22 July 2020). Written informed consent has been provided by all study participants after receiving a full explanation of the study’s purposes.

The patient dataset for the ML and cross-validation was obtained from the three-center, age- and sex-matched study, which was performed in Kemerovo (Siberia, Russia) from 1 August 2020 to 1 August 2022 and included 350 patients admitted to the ICUs of the Research Institute for Complex Issues of Cardiovascular Diseases (100 patients), Kuzbass Regional Infectious Diseases Clinical Hospital (106 patients), and Kuzbass Regional Clinical Hospital (144 patients). Criteria of inclusion were: (1) SARS-CoV-2 infection confirmed by the positive reverse transcription-polymerase chain reaction test; (2) admission to the ICU because of severe COVID-19. Nasopharyngeal swabs were collected from both nasal passages in the same tube together with oropharyngeal swabs for increasing SARS-CoV-2 concentration. RNA isolation was carried out using the RealBest Sorbitus kit (C-8848, Vector-Best, Koltsovo, Russia) and the multi-functional robotic platform Freedom EVO (Tecan, Männedorf, Switzerland). The reverse transcription-polymerase chain reaction was conducted using RealBest RNA SARS-CoV-2 kit (D-5580, Vector-Best, Koltsovo, Russia) and CFX96 Touch real-time PCR detection system (Bio-Rad, Hercules, CA, USA). At the admission to the ICU, we collected the clinical data (age, gender, and past/present medical history of arterial hypertension (AH), diabetes mellitus (DM), coronary artery disease (CAD), and/or chronic heart failure (CHF), chronic obstructive pulmonary disease (COPD) and/or asthma, and stage 3–5 chronic kidney disease (CKD)), complete blood count measurements (white blood cell count (WBC), neutrophil count (NE#), lymphocyte count (LY#), neutrophil-to-lymphocyte ratio (NLR), and platelet count (PLT)), and biochemical profiling results (blood urea nitrogen (BUN), serum creatinine (sCr), glomerular filtration rate (GFR), aspartate aminotransferase (AST), alanine aminotransferase (ALT), fasting plasma glucose (FPG), C-reactive protein (CRP), and D-dimer). GFR was calculated according to the 2021 CKD-EPI Creatinine equation. The patients were pre-matched (1:1) by age, sex, and outcome (in-hospital death or hospital discharge). The complete descriptive statistics are shown in Table 1.

**Figure 1:**
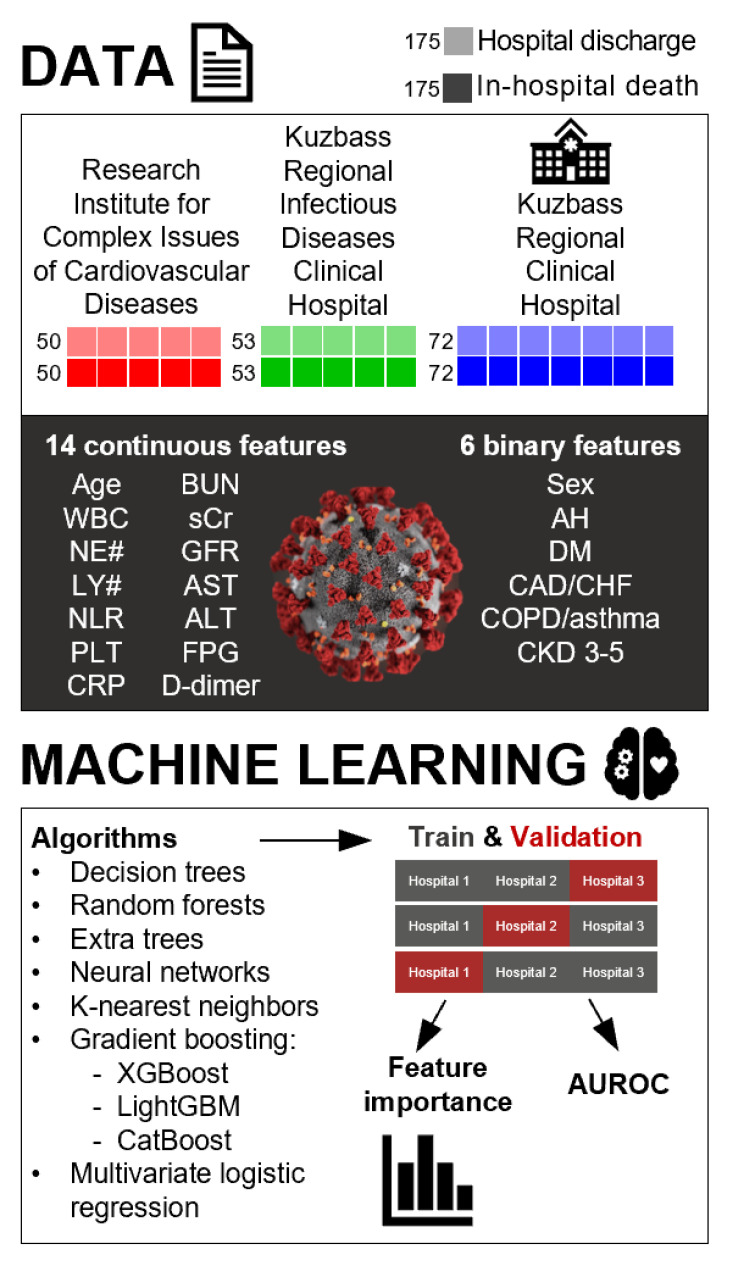
Study design. The patients (*n* = 350) enrolled from three centers (Research Institute for Complex Issues of Cardiovascular Diseases, *n* = 100; Kuzbass Regional Infectious Diseases Clinical Hospital, *n* = 106; Kuzbass Regional Clinical Hospital, *n* = 144) have been pre-matched (1:1) by age, sex (male or female), and outcome (in-hospital death or hospital discharge). This patient dataset has been employed for the ML by a number of algorithms (decision trees, random forests, extra trees, neural networks, k-nearest neighbors, gradient boosting: XGBoost, LightGBM, and CatBoost) and multivariate logistic regression as a reference. In total, we assessed 14 continuous variables (age, WBC, NE#, LY#, NLR, PLT, BUN, sCr, GFR, AST, ALT, FPG, CRP, and D-dimer) and 6 binary variables (sex and past/present medical history of AH, DM, CAD/CHF, COPD/asthma, and stage 3–5 CKD) which were measured at the admission to the ICU. The outcome was binary (in-hospital death or hospital discharge). ML and cross-validation were performed either on a general dataset (70:30 learning:cross-validation samples proportion) or on all combinations of two sub-datasets from separate hospitals (Research Institute for Complex Issues of Cardiovascular Diseases and Kuzbass Regional Infectious Diseases Clinical Hospital, *n* = 206; Research Institute for Complex Issues of Cardiovascular Diseases and Kuzbass Regional Clinical Hospital, *n* = 244; Kuzbass Regional Infectious Diseases Clinical Hospital and Kuzbass Regional Clinical Hospital, *n* = 250) using the third dataset (Kuzbass Regional Clinical Hospital, *n* = 144; Kuzbass Regional Infectious Diseases Clinical Hospital, *n* = 106; Research Institute for Complex Issues of Cardiovascular Diseases, *n* = 100, respectively) as a cross-validation sample. The efficiency of the ML algorithms and tools was evaluated by AUROC, percent of correct predictions (%sensitivity and %specificity), and the range of these parameters between the distinct study centers.

The study design is represented in Figure 1.

### 2.2. Machine Learning

To develop the ML tools for the prognosis of fatal outcomes in patients admitted to the ICU because of severe COVID-19, we used the following supervised algorithms, which are well-established in working with tabular data [7,39]:

1. Decision trees
2. Random forests
3. Extra trees
4. Neural networks (multilayer perceptron)
5. K-nearest neighbors
6. Gradient boosting algorithms:
  6.1. XGBoost
  6.2. LightGBM
  6.3. CatBoost
7. Multivariable logistic regression as a reference

The work with ML algorithms was conducted in PyCharm, an integrated development environment using Python3 programming language and NumPy, Scikit Learn, Pandas, Matplotlib, CatBoost, XGBoost, and major-supervised libraries. In the process of ML, we employed the following techniques:

1. Data preprocessing: missing data have been automatically inputted with median column values. Categorical data were replaced on the binary values, and multi categorical variables were converted into dummy variables.
2. Power normalization: as some ML algorithms are sensitive to the data distribution, we applied Power transforms, a technique for transforming numerical input or output variables to have a Gaussian or more Gaussian-like probability distribution. This approach reduced data variability and skewness. The power transformation used in our study was based on the Yeo-Johnson transformation [40].
3. Model hyperparameters were tuned by mljar-supervised “Compete” algorithm via hill climbing to fine-tune final models.
4. Multicenter cross-validation. The general dataset was divided into two the learning dataset (including two sub-datasets from separate hospitals) and the test dataset (including a sub-dataset from the remaining hospital). This procedure was performed for all combinations of learning and test datasets: (1) learning dataset: Research Institute for Complex Issues of Cardiovascular Diseases and Kuzbass Regional Infectious Diseases Clinical Hospital (*n* = 206), cross-validation dataset: Kuzbass Regional Clinical Hospital (*n* = 144); (2) learning dataset: Research Institute for Complex Issues of Cardio-vascular Diseases and Kuzbass Regional Clinical Hospital (*n* = 244), cross-validation dataset: Kuzbass Regional Infectious Diseases Clinical Hospital (*n* = 106); (3) learning dataset: Kuzbass Regional Infectious Diseases Clinical Hospital and Kuzbass Regional Clinical Hospital (*n* = 250), cross-validation dataset: Research Institute for Complex Issues of Cardiovascular Diseases (*n* = 100).
5. As the evaluation metrics, we used AUROC (the primary metric for optimization), %sensitivity, %specificity, and range (variability) of these parameters between the distinct study centers. For binary classifications, we used the default probability threshold of 0.5 (irrespective of the number of folds for cross-validation) and then calculated sensitivity and specificity. We deliberately excluded the optimization of the probability threshold to ensure an equal evaluation for each cross-validation fold. Such a custom cross-validation strategy included training on the data from the two clinics and cross-validation on the dataset from the remaining clinic, with the testing of all three possible combinations in this regard. As a consequence, we had three values (according to the number of cross-validation folds) for each of the selected metrics (sensitivity and specificity), which were obtained using a unified probability threshold (0.5). These metrics provided transparency and permitted the clinical interpretation of the algorithm efficiency.
6. Out of all models, we selected those having the highest AUROC, %sensitivity, and %specificity (9 models in total, one per each ML algorithm: decision trees, random forests, extra trees, neural networks, k-nearest neighbors, gradient boosting (XGBoost, LightGBM, and CatBoost), and multivariate logistic regression as a reference). Optimal parameters for the best models developed by each machine learning approach are provided in Table S1.
7. During the ML, we conducted a feature importance analysis using a SHAP (SHapley Additive exPlanations) technique [41], a game theoretic approach that explains the contribution of each feature to an individual predicted value (i.e., measures the impact of each factor into the output of any used ML model). For each of the selected models (*n* = 9 as described above), we quantified the feature importance within the [-1; 1] interval. Further, we applied a Predictor Screening tool of STATISTICA 13 software (TIBCO Software, Palo Alto, CA, USA).
8. In addition to PyCharm integrated development environment, we have also used STATISTICA Automated Neural Networks (SANN) tool, which automatically generates, evaluates, and exports neural networks employing a multilayer perceptron architecture according to the input variables. The screening of the most efficient neural networks has been performed manually. When using this approach, ML and cross-validation have been carried out on a general dataset (70:30 learning:cross-validation samples proportion). In addition, the most efficient neural networks underwent cross-validation on four virtual patient samples generated by bootstrapping, a statistical procedure that resamples a single dataset by repeatedly drawing samples from the source data with replacement to create a simulated dataset.

Statistical analysis was performed using GraphPad Prism 8 (GraphPad Software, San Diego, CA, USA) and STATISTICA 13 (TIBCO Software, Palo Alto, CA, USA). For descriptive statistics, data are presented as median, 25th and 75th percentiles, and range. Proportions were compared by Pearson’s chi-squared test with Yates’s correction for continuity. Two independent groups were compared by the Mann-Whitney U-test. Three independent groups were compared by the Kruskal–Wallis test with post hoc calculation of false discovery rate (FDR) by the two-stage linear step-up procedure of Benjamini, Krieger, and Yekutieli. Correlation analysis was performed using Spearman’s rank correlation coefficient. P values, or q values if FDR was applied (q values are the name given to the adjusted p values found using an optimized FDR approach), ≤0.05 were regarded as statistically significant.

## 3. Results

### 3.1. Univariate Analysis Identifies Cardiovascular Comorbidity, Immune Cell Counts, Kidney Dysfunction Markers, C-Reactive Protein, and D-Dimer Levels as the Potential Predictors of COVID-19-Related Death at the Stage of ICU Admission

To screen for the putative risk factors of COVID-19-related death at the patient admission to the ICU, we first carried out a univariate risk factor analysis (Mann-Whitney U-test). Cardiovascular comorbidity (i.e., past/present medical history of AH and CAD/CHF), immune cell parameters of complete blood count (increased WBC and NE#, reduced LY#, and elevated NLR), kidney dysfunction markers (augmented BUN and sCr and reduced GFR), and increased CRP and D-dimer were significantly associated with in-hospital death (Table 2). In contrast, diabetes mellitus, chronic obstructive pulmonary disease or asthma, stage 3–5 CKD, platelet count, alanine aminotransferase, and fasting plasma glucose did not reach statistical significance (Table 2).

**Figure 2:**
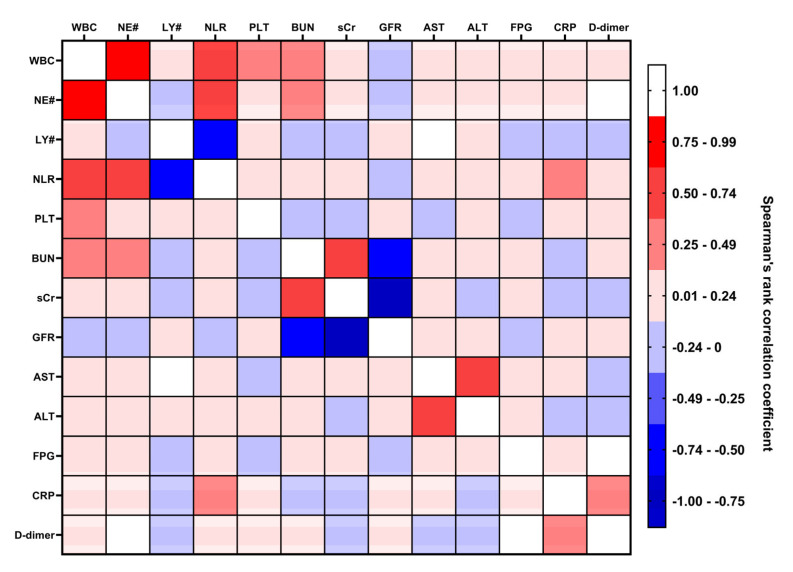
Correlation analysis (Spearman’s rank correlation coefficient) of continuous predictors. Heat map, different shades of blue (from light blue to dark blue) indicate correlation coefficients from −0.01 to −1.0, respectively; different shades of red (from pink to scarlet) indicate correlation coefficients from 0.01 to 1.0, respectively. AH—arterial hypertension, DM—diabetes mellitus, CAD—coronary artery disease. CHF—chronic heart failure, COPD—chronic obstructive pulmonary disease, CKD—chronic kidney disease, WBC—white blood cell count, NE#—neutrophil count, LY#—lymphocyte count, NLR—neutrophil-to-lymphocyte ratio, PLT—platelet count, BUN—blood urea nitrogen, sCr—serum creatinine. GFR—glomerular filtration rate, AST—aspartate aminotransferase, ALT—alanine aminotransferase, FPG—fasting plasma glucose, CRP—C-reactive protein.

Next, we searched for the associations between the predictors to facilitate their further manual selection in the process of ML. For these tasks, we performed Spearman’s rank-order correlation analysis and found statistically significant moderate correlations between BUN and WBC (r = 0.31) and BUN and NE# (r = 0.32, Table S2 and Figure 2) that suggested an association between kidney dysfunction and systemic inflammatory response. Strong correlations between WBC and NE# (r = 0.92), sCr and GFR (r = −0.89), as well correlating NLR and NE# (r = 0.67), NLR and LY# (r = −0.72), sCr and BUN (r = 0.62), and AST and ALT (r = 0.66) confirmed the technical validity of the correlation analysis and the overall data integrity (Table S2 and Figure 2).

### 3.2. Neural Networks Represent the Most Reliable and Efficient Algorithm for the Prognostication of Patients Admitted to ICU with Severe COVID-19

**Figure 3:**
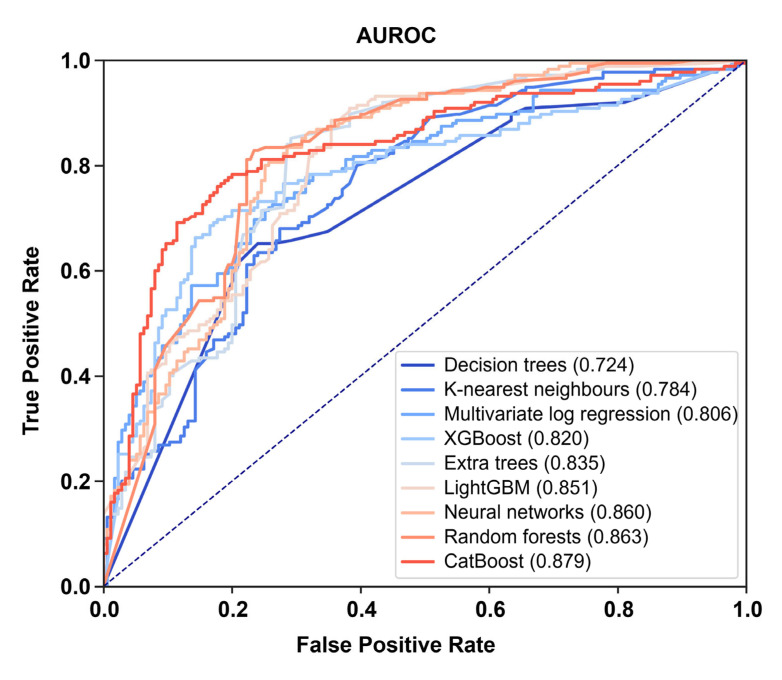
ROC curves and AUROC values for the best models developed by distinct ML algorithms.

Comparison of AUROC demonstrated significant differences in efficiency between distinct ML algorithms (Figure 3 and Table 3). The highest efficiency was showed by CatBoost (AUROC = 0.879), random forests (AUROC = 0.863), and neural networks (AUROC = 0.860), whereas decision trees (AUROC = 0.724) and K-nearest neighbors had the lowest predictive value (AUROC = 0.784, Figure 3 and Table 3). In addition to the average AUROC, we carried out multicenter cross-validation and found considerable variability of AUROC between the different centers (Table 3). The least range was demonstrated by multivariate logistic regression (reference technique, 0.014), K-nearest neighbors (0.044), XGBoost (0.058), and CatBoost (0.059, Table 3). 

**Figure 4:**
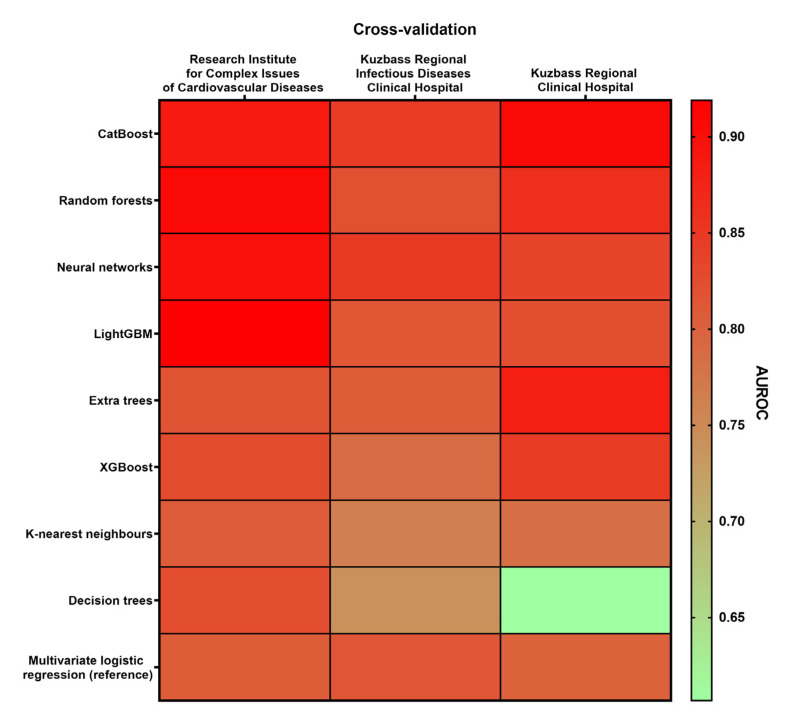
Heat map demonstrating the variability of AUROC in different combinations of learning and cross-validation samples. The color range from green to red indicates the ascending AUROC (the lowest and the highest AUROC are marked green and red, respectively). Left column: learning dataset: Kuzbass Regional Infectious Diseases Clinical Hospital and Kuzbass Regional Clinical Hospital (*n* = 250), cross-validation dataset: Research Institute for Complex Issues of Cardiovascular Diseases (*n* = 100). Central column: learning dataset: Research Institute for Complex Issues of Cardiovascular Diseases and Kuzbass Regional Clinical Hospital (*n* = 244), cross-validation dataset: Kuzbass Regional Infectious Diseases Clinical Hospital (*n* = 106). Right column: learning dataset: Research Institute for Complex Issues of Cardiovascular Diseases and Kuzbass Regional Infectious Diseases Clinical Hospital (*n* = 206), cross-validation dataset: Kuzbass Regional Clinical Hospital (*n* = 144).

Most of the models reached the highest AUROC having been learned on the samples recruited in the Kuzbass Regional Infectious Diseases Clinical Hospital and Kuzbass Regional Clinical Hospital (*n* = 250) and cross-validated on the sample enrolled in the Research Institute for Complex Issues of Cardiovascular Diseases (*n* = 100), underscoring the substantial heterogeneity between the study centers (Figure 4).

**Figure 5:**
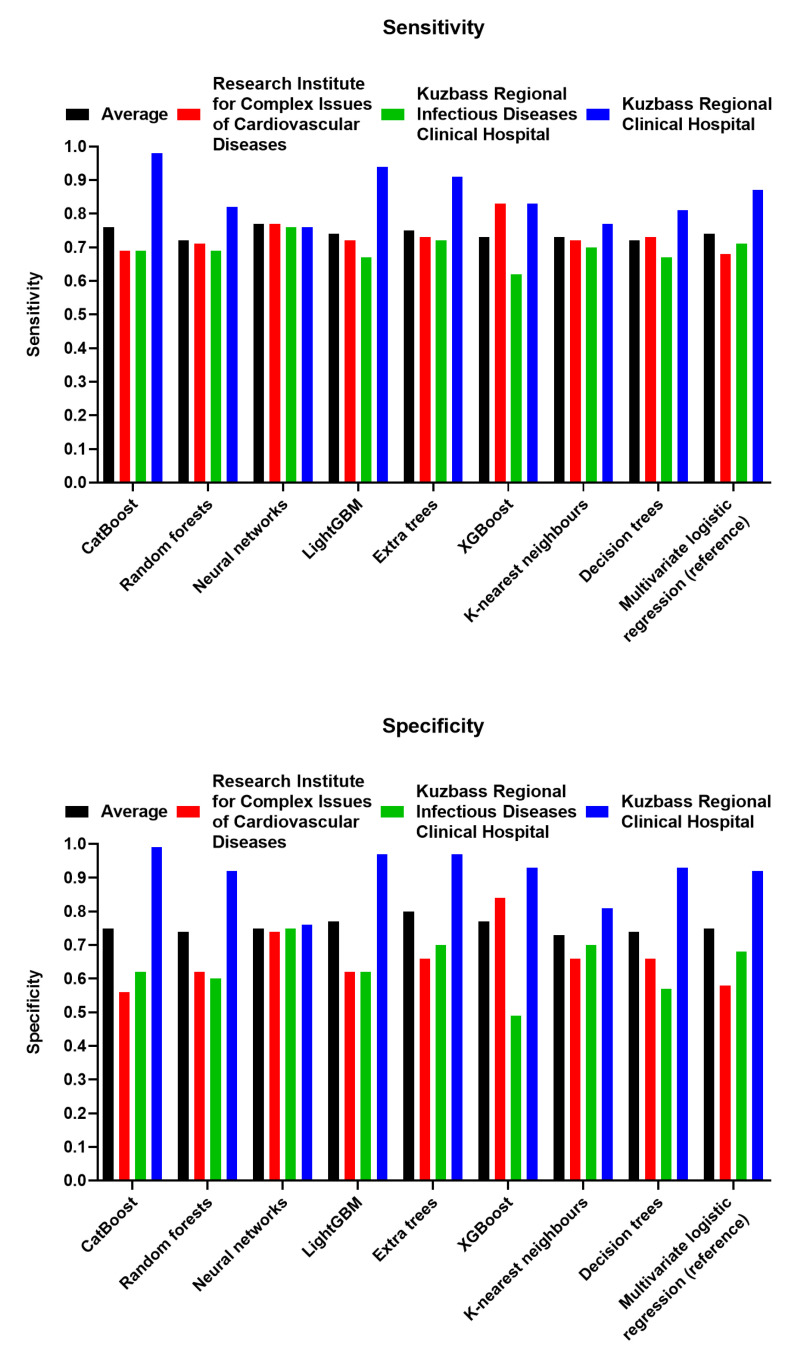
Sensitivity (**top**) and specificity (**bottom**) of the employed ML algorithms. Black color: average value across three centers, red color: Research Institute for Complex Issues of Cardiovascular Diseases (*n* = 100); green color: Kuzbass Regional Infectious Diseases Clinical Hospital (*n* = 106); blue color: Kuzbass Regional Clinical Hospital (*n* = 144).

Besides AUROC, the clinically relevant model must demonstrate equal or close sensitivity and specificity. Both sensitivity and specificity were significantly higher when learning predictive models on the samples collected from the Research Institute for Complex Issues of Cardiovascular Diseases and Kuzbass Regional Infectious Diseases Clinical Hospital (*n* = 206) and cross-validating them on the sample obtained in Kuzbass Regional Clinical Hospital (*n* = 144), confirming the heterogeneity between the samples enrolled in different centers (Figure 5). Among all ML algorithms, neural networks had the highest average sensitivity and third-rank average specificity, suggesting potentially high predictive value as compared to other algorithms (Figure 5 and Table 4). Importantly, neural networks showed the highest robustness in relation to the sample heterogeneity across different study centers (i.e., the least range between the sensitivities and specificities obtained in distinct centers, Table 4).

We have further screened the ensembles of neural networks using the SANN tool of the STATISTICA software to find the combination of the most sensitive and specific predictors and the most efficient instrument to predict the fatal outcome in patients admitted to the ICU because of severe COVID-19. Manual screening identified CAD/CHF, stage 3–5 CKD, BUN, and CRP as the best composition of the predictors (Table S3). Out of manually selected 15 neural networks with a percent of correct predictions >80% and AUROC >0.8, four showed AUROC of ≥ 0.85 (0.850, 0.853, 0.861, and 0.866), two showed sensitivity >90% (#9 and #12), and one demonstrated sensitivity and specificity >80% (#1) at primary cross-validation (30% of the general dataset, Table S3). Additional cross-validation of this neural networks ensemble using the bootstrapping approach (4 virtual cross-validation samples) testified to their high predictive value, as a neural network with AUROC = 0.866 at primary cross-validation had AUROC = 0.849 and almost 80% of correct predictions as average on the bootstrapping samples (Table S3). Other neural networks developed by the manual predictor screening approach also showed AUROC ≥ 0.8, and 10/15 of them demonstrated >80% of correct predictions after the bootstrapping cross-validation (Table S3).

Next, we ranked all predictors using the Predictor Screening tool of the STATISTICA software. Among all, CAD/CHF, CRP, LY#, and NLR were suggested as having the highest impact on mortality (information value > 0.3 and Cramer’s V > 0.25, Table 5). However, only one of the top 10 neural networks selected by the automated predictor screening (#2, AUROC = 0.824, Table S4) reached the least AUROC of neural networks developed with the manual predictor screening (#6, 0.823, #6, Table S3) at primary cross-validation and none of them yielded AUROC > 0.8 or 80% of correct predictions at bootstrapping cross-validation (Table S4). 

The combination of manual and automated screening predictors (CAD/CHF, stage 3–5 CKD, BUN, CRP, LY#, and NLR) did not improve the sensitivity and specificity of generated neural networks, as none of them contested AUROC of 0.85 at primary cross-validation (Table S5). To summarize, neural networks were the most efficient (in particular, the most sensitive) ML algorithm for predicting the fatal outcome in age- and sex-matched patients with severe COVID-19 admitted to the ICU. Another advantage of neural networks was their robustness to the heterogeneity between the samples enrolled in distinct study centers. The AUROC of some neural networks exceeded 0.85 at primary cross-validation and reached almost similar values at virtual samples generated from the existing validation dataset by a bootstrapping procedure. The most valuable predictors at the time of the ICU admission were CAD/CHF, stage 3–5 CKD, BUN, and CRP. As all of these parameters can be measured and analyzed rapidly, and BUN and CRP are relatively inexpensive, the such composition of the predictors may be highly efficient for the triage of patients with severe COVID-19 in the ICUs if applied in a neural network context.

## 4. Discussion

Albeit a lot of ML tools, including neural networks, for the prediction of COVID-19 outcomes have been developed and implemented [42,43,44,45,46], multicenter, age- and sex-matched studies are still rarely encountered. Albeit most of the studies focused on chest X-ray and computed tomography images [5,6,7], the use of routine clinical data (e.g., existing comorbidities or past/present medical history of cardiovascular events) and basic hematological and biochemical parameters embedded into the decision support system offers an opportunity of the rapid triage of SARS-CoV-2-positive patients with regards to their admission to the ICU or choice of treatment modalities within the ICU. The use of ML instruments removes subjectivity from the triage process as compared to the analysis of symptoms or complaints. However, the development of efficient AI tools is largely dependent on the study sample (including the treatment protocols, which vary between the hospitals) and the prevalence of the risk factors in the respective communities and patient cohorts. Further, ascending age and skewed recruitment of male or female patients inevitably create a bias as a number of comorbidities are more frequent in the elderly or in men or women. In order to avoid the aforementioned drawbacks, ML studies must be performed in distinct centers, and patient cohorts should be matched by age and sex in order to reveal the independent pathophysiological factors defining the risk of the fatal outcome.

The efficiency of ML classifier algorithms is typically estimated using AUROC (an ultimate metric combining sensitivity and specificity), sensitivity and specificity separately, and range between AUROC, sensitivities, and specificities obtained in distinct study centers. The latter metrics can be considered as a measure of robustness, as such support decision systems are designed to be applied in different clinics, and, therefore, they must avoid overfitting. In our study, we focused both on the selection of the best ML algorithm and on the development of best-in-class AI tools. The two-stage design of the study can be described as follows: first, we performed 3-fold cross-validation (where each fold represents one center or comparison) for all tested ML algorithms (decision trees, random forests, extra trees, neural networks, k-nearest neighbors, and gradient boosting: XGBoost, LightGBM, and CatBoost), using multivariate logistic regression as a reference. Then, we selected the best ML tool in terms of average sensitivity, specificity, and robustness in relation to the sample heterogeneity across different study centers. To find a combination of the most sensitive and specific predictors and to develop efficient ML tools to predict the fatal outcome, we applied a 70:30 split validation in the second stage of our study.

Screening of broadly established ML algorithms (decision trees, random forests, extra trees, neural networks, k-nearest neighbors, and gradient boosting: XGBoost, LightGBM, and CatBoost) and multivariate logistic regression as a reference revealed that neural networks exhibit the highest sensitivity, good specificity, and perfect robustness (the negligible range of sensitivities and specificities obtained in different study centers). Moreover, selected neural networks were able to exceed AUROC of 0.85, 90% sensitivity, and 80% specificity. The most valuable predictors included the past/present medical history of CAD/CHF and/or stage 3–5 CKD, BUN, and CRP, and therefore the cost of such screening analysis does not exceed 15 USD or EUR that is important for the medical economy, in particular in the middle- and low-income countries. Having pinpointed the age- and sex-independent importance of cardiovascular and renal comorbidities in the prediction of COVID-19 fatal outcomes in the ICU setting, we suggest that incorporation of other clinical factors such as age or body mass index into the neural networks may further improve their sensitivity and specificity, as the pathophysiological background of the such predictive model has been clarified in this study. In addition, the finding the specific endothelial dysfunction marker, which is currently lacking [47,48,49], may finalize the model, as pro-thrombotic and pro-inflammatory activation of endothelial cells represents a characteristic feature of COVID-19 [50,51,52,53].

In comparison with neural networks, random forests and CatBoost (one of the gradient boosting algorithms) showed higher average AUROC but also a considerably wider range in sensitivity and specificity, overall suggesting lower robustness. This observation emphasizes the importance of conducting multicenter instead of single-center studies. However, most of the existing predictive models have been developed in single-center studies, therefore suffering from a high risk of bias and also sharing common shortcomings such as inadequate sample size, poor or unclear handling of missing data, and weak cross-validation [18,54]. Even the multicenter studies often do not report the variability between the centers, although some of them did [21,22,24,55]. Having ranked all machine learning algorithms by sensitivity and specificity, we found that extra trees had an average rank similar to neural networks and higher specificity, whilst neural networks demonstrated higher sensitivity that is presumably more important in the context of prompt triage in the ICU setting.

The contribution of cardiovascular comorbidity (past/present medical history of coronary artery disease and chronic heart failure) to the successful prediction of COVID-19-related death after the patient admission to ICU has been reported as controversial, as some of the studies did not evaluate its impact as significant [55] whereas other reports documented its prognostic importance [56,57,58,59], similar to our results. In contrast, the impact of renal comorbidity on the mortality from COVID-19 has been well described [60,61,62,63], and the prognostic importance of CRP [21,22,24,64,65,66] and BUN [22,23,25,67,68,69,70,71] has been clearly reported as evident in multiple ML studies and meta-analyses.

Despite the fact we applied a multicenter enrollment that permitted primary cross-validation between the study centers and performed a bootstrapping procedure for additional virtual cross-validation when using a general dataset, and patient samples with distinct outcomes were age- and sex-matched, our study still has certain shortcomings. First, as we recruited patients from 2020 (when anti-SARS-CoV-2 vaccines have not been implemented, as Sputnik V was deployed on 27 November 2020 and widely distributed since January 2021) to 2022 (when the vaccination rate exceeded 50%), the vaccination status could impact the relative contribution of the predictors. Second, the sample size (*n* = 350) was limited because of age and sex-matching necessity, and resampling of patients regardless of age can increase the sensitivity and specificity of our neural networks. Yet, this can be considered as an aim for further study. Third, although all datasets included age- and sex-matched patients, the total number of matched pairs was limited (*n* = 175). An independent validation of the proposed models would be important for the unbiased evaluation of machine learning algorithms’ performance. Fourth, all study centers were located in the same geographic region, restricting the generalizability of the developed models.

## 5. Conclusions

We conclude that neural networks represent an optimal ML algorithm to predict the fatal outcome in age- and sex-matched patients with severe COVID-19 admitted to the ICU, as they showed the highest sensitivity (>90%), sufficient specificity (>80%), good AUROC (>0.86 at primary cross-validation and ≈0.95 at virtual cross-validation if using a bootstrapping technique), and excellent robustness to the heterogeneity between the study centers. The most valuable predictors of COVID-19-related death at the time of the ICU admission were CAD/CHF, stage 3–5 CKD, BUN, and CRP. We suggest that this composition of the predictors might be applicable for the expedient triage of patients with severe COVID-19 in the ICUs if embedded into the neural network (as has been shown in the study).

## Figures and Tables

**Table 1:** Descriptive statistics across three centers participating in the study.

Feature	Research Institute for Complex Issues of Cardiovascular Diseases (*n* = 100)	Kuzbass Regional Infectious Diseases Clinical Hospital (*n* = 106)	Kuzbass Regional Clinical Hospital (*n* = 144)	FDR-Corrected *p* Value	Average (*n* = 350)
Clinical data					
Sex, M/F, *n* (%)	50/50 (50.00%/50.00%)	53/53 (50.00%/50.00%)	72/72 (50.00%/50.00%)	1.00	175/175 (50.00%/50.00%)
Age, years, Me [IQR]	73.00 [67.00–80.75]	68.50 [62.75–79.00]	64.00 [56.25–69.00]	0.0001	68.00 [61.00–75.00]
AH, *n* (%)	98/100 (98.00%)	90/106 (84.90%)	115/144 (79.86%)	0.0001	297/350 (84.86%)
DM, *n* (%)	35/100 (35.00%)	36/106 (33.96%)	63/144 (43.75%)	0.21	131/350 (37.43%)
CAD/CHF, *n* (%)	86/100 (86.00%)	55/106 (51.89%)	91/144 (63.19%)	0.0001	232/350 (66.29%)
COPD/asthma, *n* (%)	12/100 (12.00%)	7/106 (6.60%)	17/144 (11.80%)	0.33	36/350 (10.29%)
Stage 3–5 CKD, *n* (%)	34/100 (34.00%)	10/106 (9.43%)	38/144 (26.39%)	0.0001	82/350 (23.43%)
Complete blood count measurements					
WBC, × 10^9^/L, Me [IQR]	7.30 [5.25–11.68]	8.55 [5.50–13.45]	10.75 [7.82–14.20]	0.0001	9.10 [6.00–12.90]
NE#, × 10^9^/L, Me [IQR]	5.40 [3.25–9.10]	7.55 [4.30–11.50]	9.20 [6.60–12.40]	0.0001	7.80 [4.50–11.30]
LY#, × 10^9^/L, Me [IQR]	1.20 [0.60–1.90]	0.90 [0.40–1.20]	0.80 [0.50–1.20]	0.0003	0.90 [0.50–1.30]
NLR, Me [IQR]	4.75 [2.20–12.40]	9.40 [5.30–17.55]	11.60 [6.87–17.60]	0.0001	9.10 [4.70–16.33]
PLT, × 10^9^/L, Me [IQR]	193.5 [154.8–266.8]	188.5 [156.0–253.5]	239.5 [178.3–297.5]	0.0003	216.0 [160.0–277.0]
Biochemical profiling					
BUN, mmol/L, Me [IQR]	6.95 [5.97–9.32]	7.80 [5.60–13.83]	8.45 [6.00–12.05]	0.09	7.80 [5.90–11.75]
sCr, µmol/L, Me [IQR]	87.00 [74.00–107.00]	97.00 [76.75–126.80]	78.50 [65.25–106.50]	0.0001	85.00 [69.75–112.30]
GFR (CKD-EPI), mL/min/1.73 m^2^, Me [IQR]	69.00 [51.00–85.00]	60.00 [44.75–79.50]	86.50 [57.25–98.75]	0.0001	73.00 [50.75–93.00]
AST, U/L, Me [IQR]	22.50 [18.25–35.00]	39.00 [30.75–58.00]	42.00 [27.00–66.75]	0.0001	36.00 [25.00–55.25]
ALT, U/L, Me [IQR]	21.50 [16.00–30.00]	31.00 [25.75–47.00]	38.50 [22.25–60.75]	0.0001	30.00 [20.00–48.25]
FPG, mmol/L, Me [IQR]	6.20 [5.30–7.37]	7.10 [5.57–11.63]	7.20 [5.60–9.45]	0.0017	6.70 [5.50–8.92]
CRP, mg/L, Me [IQR]	52.00 [17.25–165.00]	41.50 [13.00–109.30]	101.00 [47.75–164.80]	0.0001	68.50 [22.75–140.00]
D-dimer, ng/mL, Me [IQR]	2685 [856–6701]	1011 [325–1379]	2974 [1406–5528]	0.0001	1802 [840–4320]
Outcome					
In-hospital death/hospital discharge, n (%)	50/50 (50.00%/50.00%)	53/53 (50.00%/50.00%)	72/72 (50.00%/50.00%)	1.00	175/175 (50.00%/50.00%)

M—male, F—female, FDR—false discovery rate, Me—median, IQR—interquartile range, AH—arterial hypertension, DM—diabetes mellitus, CAD—coronary artery disease. CHF—chronic heart failure, COPD—chronic obstructive pulmonary disease, CKD—chronic kidney disease, WBC—white blood cell count, NE#—neutrophil count, LY#—lymphocyte count, NLR—neutrophil-to-lymphocyte ratio, PLT—platelet count, BUN—blood urea nitrogen, sCr—serum creatinine. GFR—glomerular filtration rate, AST—aspartate aminotransferase, ALT—alanine aminotransferase, FPG—fasting plasma glucose, CRP—C-reactive protein.

**Table 2:** Descriptive statistics and univariate risk factor analysis in patients with adverse (in-hospital death) and favorable (hospital discharge) outcomes.

Feature	In-Hospital Death (*n* = 175)	Hospital Discharge (*n* = 175)	*p* Value
Clinical data			
Sex, M/F, *n* (%)	79/96 (45.14%/54.86%)	79/96 (45.14%/54.86%)	N/A
Age, years, Me [IQR]	68.00 [61.00–75.00]	68.00 [61.00–75.00]	N/A
AH, *n* (%)	161/175 (92.00%)	142/175 (81.14%)	0.003
DM, *n* (%)	70/175 (40.00%)	64/175 (36.57%)	0.51
CAD/CHF, *n* (%)	153/175 (87.43%)	79/175 (45.14%)	0.0001
COPD/asthma, *n* (%)	16/175 (9.14%)	20/175 (11.43%)	0.48
Stage 3–5 CKD, *n* (%)	45/175 (25.71%)	37/175 (21.14%)	0.31
Complete blood count measurements			
WBC, × 10^9^/L, Me [IQR]	10.00 [6.70–14.30]	8.70 [5.60–11.70]	0.004
NE#, × 10^9^/L, Me [IQR]	8.70 [5.50–12.80]	6.80 [3.80–9.90]	0.0001
LY#, × 10^9^/L, Me [IQR]	0.70 [0.50–1.20]	1.00 [0.70–1.50]	0.0004
NLR, Me [IQR]	11.40 [6.80–20.60]	6.90 [3.10–13.60]	0.0001
PLT, × 10^9^/L, Me [IQR]	208.0 [156.0–269.0]	219.0 [167.0–284.0]	0.09
Biochemical profiling			
BUN, mmol/L, Me [IQR]	8.30 [6.50–13.10]	7.40 [5.60–10.60]	0.007
sCr, µmol/L, Me [IQR]	89.0 [72.0–120.0]	83.0 [68.0–107.0]	0.014
GFR (CKD-EPI), mL/min/1.73 m^2^, Me [IQR]	69.00 [48.00–90.00]	75.00 [54.00–94.00]	0.05
AST, U/L, Me [IQR]	37.00 [25.00–61.00]	35.00 [23.00–50.00]	0.07
ALT, U/L, Me [IQR]	28.00 [20.00–46.00]	30.00 [20.00–50.00]	0.54
FPG, mmol/L, Me [IQR]	7.10 [5.50–9.90]	6.40 [5.50–8.30]	0.13
CRP, mg/L, Me [IQR]	101.0 [50.0–164.0]	37.0 [10.0–109.0]	0.0001
D-dimer, ng/mL, Me [IQR]	2770 [1194–5001]	1263 [565–3463]	0.0001

M—male, F—female, Me—median, IQR—interquartile range, AH—arterial hypertension, DM—diabetes mellitus, CAD—coronary artery disease. CHF—chronic heart failure, COPD—chronic obstructive pulmonary disease, CKD—chronic kidney disease, WBC—white blood cell count, NE#—neutrophil count, LY#—lymphocyte count, NLR—neutrophil-to-lymphocyte ratio, PLT—platelet count, BUN—blood urea nitrogen, sCr—serum creatinine. GFR—glomerular filtration rate, AST—aspartate aminotransferase, ALT—alanine aminotransferase, FPG—fasting plasma glucose, CRP—C-reactive protein.

**Table 3:** Comparison of AUROC values for the best models developed by distinct ML algorithms.

Machine Learning Algorithm	AUROC				
	Research Institute for Complex Issues of Cardiovascular Diseases (*n* = 100)	Kuzbass Regional Infectious Diseases Clinical Hospital (*n* = 106)	Kuzbass Regional Clinical Hospital (*n* = 144)	Average	Range
Decision trees	0.824	0.740	0.607	0.724	0.217
Random forests	0.908	0.821	0.861	0.863	0.087
Extra trees	0.817	0.806	0.882	0.835	0.076
Neural networks	0.898	0.849	0.834	0.860	0.064
K-nearest neighbors	0.807	0.763	0.782	0.784	0.044
XGBoost	0.827	0.787	0.845	0.820	0.058
LightGBM	0.919	0.812	0.822	0.851	0.107
CatBoost	0.887	0.846	0.905	0.879	0.059
Multivariate logistic regression (reference)	0.805	0.813	0.799	0.806	0.014

**Table 4:** Ranking of the ML algorithms according to their sensitivity and specificity. The least rank means the highest sensitivity (sens.) or specificity (spec.).

Machine Learning Algorithm	Average Sens.	Average Spec.	Range (Sens.)	Range (Spec.)	Rank (Average Sens.)	Rank (Average Spec.)	Rank (Total)
Decision trees	0.72	0.74	0.14	0.36	6	4	10
Random forests	0.72	0.74	0.13	0.31	6	4	10
Extra trees	0.75	0.80	0.19	0.31	3	1	4
Neural networks	0.77	0.75	0.01	0.02	1	3	4
K-nearest neighbors	0.73	0.73	0.07	0.15	5	5	10
XGBoost	0.73	0.77	0.21	0.44	5	2	7
LightGBM	0.74	0.77	0.26	0.35	4	2	6
CatBoost	0.76	0.75	0.28	0.43	2	3	5
Multivariate logistic regression (reference)	0.74	0.75	0.18	0.34	4	3	7

**Table 5:** Ranking of the predictors by the Predictor Screening tool of the STATISTICA software.

Predictor	Gini	Information Value	Cramer’s V
CAD/CHF	0.40	0.90	0.45
CRP	0.41	0.84	0.41
LY#	0.45	0.39	0.30
NLR	0.46	0.36	0.29
D-dimer	0.47	0.26	0.25
FPG	0.47	0.20	0.22
NE#	0.48	0.18	0.21
PLT	0.48	0.16	0.18
WBC	0.48	0.13	0.18
BUN	0.49	0.11	0.16
AH	0.49	0.11	0.16
GFR	0.49	0.11	0.16
sCr	0.49	0.10	0.15
AST	0.49	0.08	0.14
ALT	0.50	0.04	0.10
Stage 3–5 CKD	0.50	0.01	0.05
COPD/asthma	0.50	0.01	0.04
DM	0.50	0.00	0.04

CAD—coronary artery disease, CHF—chronic heart failure, CRP—C-reactive protein, LY#—lymphocyte count, NLR—neutrophil-to-lymphocyte ratio, FPG—fasting plasma glucose, NE#—neutrophil count, PLT—platelet count, WBC—white blood cell count, BUN—blood urea nitrogen, AH—arterial hypertension, GFR—glomerular filtration rate, sCr—serum creatinine, AST—aspartate aminotransferase, ALT—alanine aminotransferase, CKD—chronic kidney disease, COPD—chronic obstructive pulmonary disease, DM—diabetes mellitus.

## Data Availability

The data presented in this study are available on request from the corresponding author. The data are not publicly available due to privacy reasons.

## Supplementary Materials

The following supporting information can be downloaded at: https://www.mdpi.com/article/10.3390/jcdd10020039/s1, Table S1: Optimal parameters for the best models with regards to each machine learning approach; Table S2: Correlation matrix of continuous predictors of COVID-19-related death in patients who has been admitted to the intensive care unit (ICU) because of severe COVID-19; Table S3: Classification summary and AUROC of neural networks for the prediction of fatal outcome in patients admitted to the ICU with severe COVID-19 (merged dataset from three centers). Manually selected predictors (CAD/CHF, stage 3–5 CKD, BUN, and CRP); Table S4: Classification summary and AUROC of neural networks for the prediction of fatal outcome in patients admitted to the ICU with severe COVID-19 (merged dataset from three centers). Predictors identified by Predictor Screening tool of STATISTICA software (CAD/CHF, LY#, NLR, and CRP); Table S5: Classification summary and AUROC of neural networks for the prediction of fatal outcome in patients admitted to the ICU with severe COVID-19 (merged dataset from three centers). Predictors combined from the manual screening and Predictor Screening tool of STATISTICA software (CAD/CHF, stage 3–5 CKD, BUN, LY#, NLR, and CRP).

## References

[1] WHO Coronavirus (COVID-19) Dashboard. https://covid19.who.int/.

[2] Kamran F., Tang S., Otles E., McEvoy D.S., Saleh S.N., Gong J., Li B.Y., Dutta S., Liu X., Medford R.J. (2022). Early identification of patients admitted to hospital for COVID-19 at risk of clinical deterioration: Model development and multisite external validation study. BMJ.

[3] Yadaw A.S., Li Y.C., Bose S., Iyengar R., Bunyavanich S., Pandey G. (2020). Clinical features of COVID-19 mortality: Development and validation of a clinical prediction model. Lancet Digit. Health.

[4] Rasmy L., Nigo M., Kannadath B.S., Xie Z., Mao B., Patel K., Zhou Y., Zhang W., Ross A., Xu H. (2022). Recurrent neural network models (CovRNN) for predicting outcomes of patients with COVID-19 on admission to hospital: Model development and validation using electronic health record data. Lancet Digit. Health.

[5] Jiao Z., Choi J.W., Halsey K., Tran T.M.L., Hsieh B., Wang D., Eweje F., Wang R., Chang K., Wu J. (2021). Prognostication of patients with COVID-19 using artificial intelligence based on chest x-rays and clinical data: A retrospective study. Lancet Digit. Health.

[6] Mei X., Lee H.C., Diao K.Y., Huang M., Lin B., Liu C., Xie Z., Ma Y., Robson P.M., Chung M. (2020). Artificial intelligence-enabled rapid diagnosis of patients with COVID-19. Nat. Med..

[7] Meraihi Y., Gabis A.B., Mirjalili S., Ramdane-Cherif A., Alsaadi F.E. (2022). Machine Learning-Based Research for COVID-19 Detection, Diagnosis, and Prediction: A Survey. SN Comput. Sci..

[8] Ustebay S., Sarmis A., Kaya G.K., Sujan M. (2022). A comparison of machine learning algorithms in predicting COVID-19 prognostics. Intern. Emerg. Med..

[9] Fernandes F.T., de Oliveira T.A., Teixeira C.E., Batista A.F.M., Dalla Costa G., Chiavegatto Filho A.D.P. (2021). A multipurpose machine learning approach to predict COVID-19 negative prognosis in São Paulo, Brazil. Sci. Rep..

[10] Jimenez-Solem E., Petersen T.S., Hansen C., Hansen C., Lioma C., Igel C., Boomsma W., Krause O., Lorenzen S., Selvan R. (2021). Developing and validating COVID-19 adverse outcome risk prediction models from a bi-national European cohort of 5594 patients. Sci. Rep..

[11] Guan X., Zhang B., Fu M., Li M., Yuan X., Zhu Y., Peng J., Guo H., Lu Y. (2021). Clinical and inflammatory features based machine learning model for fatal risk prediction of hospitalized COVID-19 patients: Results from a retrospective cohort study. Ann. Med..

[12] An C., Lim H., Kim D.W., Chang J.H., Choi Y.J., Kim S.W. (2020). Machine learning prediction for mortality of patients diagnosed with COVID-19: A nationwide Korean cohort study. Sci. Rep..

[13] Kukar M., Gunčar G., Vovko T., Podnar S., Černelč P., Brvar M., Zalaznik M., Notar M., Moškon S., Notar M. (2021). COVID-19 diagnosis by routine blood tests using machine learning. Sci. Rep..

[14] Thell R., Zimmermann J., Szell M., Tomez S., Eisenburger P., Haugk M., Kreil A., Spiel A., Blaschke A., Klicpera A. (2021). Standard blood laboratory values as a clinical support tool to distinguish between SARS-CoV-2 positive and negative patients. Sci. Rep..

[15] AlJame M., Imtiaz A., Ahmad I., Mohammed A. (2021). Deep forest model for diagnosing COVID-19 from routine blood tests. Sci. Rep..

[16] Abayomi-Alli O.O., Damaševičius R., Maskeliūnas R., Misra S. (2022). An Ensemble Learning Model for COVID-19 Detection from Blood Test Samples. Sensors.

[17] Zuin G., Araujo D., Ribeiro V., Seiler M.G., Prieto W.H., Pintão M.C., Dos Santos Lazari C., Granato C.F.H., Veloso A. (2022). Prediction of SARS-CoV-2-positivity from million-scale complete blood counts using machine learning. Commun. Med..

[18] Bottino F., Tagliente E., Pasquini L., Napoli A.D., Lucignani M., Figà-Talamanca L., Napolitano A. (2021). COVID Mortality Prediction with Machine Learning Methods: A Systematic Review and Critical Appraisal. J. Pers. Med..

[19] Araújo D.C., Veloso A.A., Borges K.B.G., Carvalho M.D.G. (2022). Prognosing the risk of COVID-19 death through a machine learning-based routine blood panel: A retrospective study in Brazil. Int. J. Med. Inform..

[20] Hu C., Liu Z., Jiang Y., Shi O., Zhang X., Xu K., Suo C., Wang Q., Song Y., Yu K. (2021). Early prediction of mortality risk among patients with severe COVID-19, using machine learning. Int. J. Epidemiol..

[21] Vaid A., Somani S., Russak A.J., De Freitas J.K., Chaudhry F.F., Paranjpe I., Johnson K.W., Lee S.J., Miotto R., Richter F. (2020). Machine Learning to Predict Mortality and Critical Events in a Cohort of Patients With COVID-19 in New York City: Model Development and Validation. J. Med. Internet Res..

[22] Bertsimas D., Lukin G., Mingardi L., Nohadani O., Orfanoudaki A., Stellato B., Wiberg H., Gonzalez-Garcia S., Parra-Calderón C.L., Robinson K. (2020). COVID-19 mortality risk assessment: An international multi-center study. PLoS ONE.

[23] Booth A.L., Abels E., McCaffrey P. (2021). Development of a prognostic model for mortality in COVID-19 infection using machine learning. Mod. Pathol..

[24] Ko H., Chung H., Kang W.S., Park C., Kim D.W., Kim S.E., Chung C.R., Ko R.E., Lee H., Seo J.H. (2020). An Artificial Intelligence Model to Predict the Mortality of COVID-19 Patients at Hospital Admission Time Using Routine Blood Samples: Development and Validation of an Ensemble Model. J. Med. Internet Res..

[25] Jamshidi E., Asgary A., Tavakoli N., Zali A., Setareh S., Esmaily H., Jamaldini S.H., Daaee A., Babajani A., Sendani Kashi M.A. (2022). Using Machine Learning to Predict Mortality for COVID-19 Patients on Day 0 in the ICU. Front. Digit. Health.

[26] Afrash M.R., Shanbehzadeh M., Kazemi-Arpanahi H. (2022). Predicting Risk of Mortality in COVID-19 Hospitalized Patients using Hybrid Machine Learning Algorithms. J. Biomed. Phys. Eng..

[27] Li S., Lin Y., Zhu T., Fan M., Xu S., Qiu W., Chen C., Li L., Wang Y., Yan J. (2021). Development and external evaluation of predictions models for mortality of COVID-19 patients using machine learning method. Neural Comput. Appl..

[28] Farr R.J., Rootes C.L., Rowntree L.C., Nguyen T.H.O., Hensen L., Kedzierski L., Cheng A.C., Kedzierska K., Au G.G., Marsh G.A. (2021). Altered microRNA expression in COVID-19 patients enables identification of SARS-CoV-2 infection. PLoS Pathog..

[29] Fraser D.D., Patterson E.K., Slessarev M., Gill S.E., Martin C., Daley M., Miller M.R., Patel M.A., Dos Santos C.C., Bosma K.J. (2020). Endothelial Injury and Glycocalyx Degradation in Critically Ill Coronavirus Disease 2019 Patients: Implications for Microvascular Platelet Aggregation. Crit. Care Explor..

[30] Fraser D.D., Cepinskas G., Slessarev M., Martin C., Daley M., Miller M.R., O’Gorman D.B., Gill S.E., Patterson E.K., Dos Santos C.C. (2020). Inflammation Profiling of Critically Ill Coronavirus Disease 2019 Patients. Crit. Care Explor..

[31] Fraser D.D., Slessarev M., Martin C.M., Daley M., Patel M.A., Miller M.R., Patterson E.K., O’Gorman D.B., Gill S.E., Wishart D.S. (2020). Metabolomics Profiling of Critically Ill Coronavirus Disease 2019 Patients: Identification of Diagnostic and Prognostic Biomarkers. Crit. Care Explor..

[32] Fraser D.D., Cepinskas G., Patterson E.K., Slessarev M., Martin C., Daley M., Patel M.A., Miller M.R., O’Gorman D.B., Gill S.E. (2020). Novel Outcome Biomarkers Identified with Targeted Proteomic Analyses of Plasma from Critically Ill Coronavirus Disease 2019 Patients. Crit. Care Explor..

[33] Byeon S.K., Madugundu A.K., Garapati K., Ramarajan M.G., Saraswat M., Kumar-M P., Hughes T., Shah R., Patnaik M.M., Chia N. (2022). Development of a multiomics model for identification of predictive biomarkers for COVID-19 severity: A retrospective cohort study. Lancet Digit. Health.

[34] Fraser D.D., Cepinskas G., Slessarev M., Martin C.M., Daley M., Patel M.A., Miller M.R., Patterson E.K., O’Gorman D.B., Gill S.E. (2021). Detection and Profiling of Human Coronavirus Immunoglobulins in Critically Ill Coronavirus Disease 2019 Patients. Crit. Care Explor..

[35] Hahm C.R., Lee Y.K., Oh D.H., Ahn M.Y., Choi J.P., Kang N.R., Oh J., Choi H., Kim S. (2021). Factors Associated with Worsening Oxygenation in Patients with Non-severe COVID-19 Pneumonia. Tuberc. Respir. Dis..

[36] Juneja G.K., Castelo M., Yeh C.H., Cerroni S.E., Hansen B.E., Chessum J.E., Abraham J., Cani E., Dwivedi D.J., Fraser D.D. (2021). Biomarkers of coagulation, endothelial function, and fibrinolysis in critically ill patients with COVID-19: A single-center prospective longitudinal study. J. Thromb. Haemost..

[37] Soya E., Ekenel N., Savas R., Toprak T., Bewes J., Doganay O. (2022). Pixel-based analysis of pulmonary changes on CT lung images due to COVID-19 pneumonia. J. Clin. Imaging Sci..

[38] Attia Z.I., Kapa S., Dugan J., Pereira N., Noseworthy P.A., Jimenez F.L., Cruz J., Carter R.E., DeSimone D.C., Signorino J. (2021). Rapid Exclusion of COVID Infection with the Artificial Intelligence Electrocardiogram. Mayo Clin. Proc..

[39] Uddin S., Khan A., Hossain M.E., Moni M.A. (2019). Comparing different supervised machine learning algorithms for disease prediction. BMC Med. Inform. Decis. Mak..

[40] Yeo I.-K. (2000). A new family of power transformations to improve normality or symmetry. Biometrika.

[41] Ning Y., Ong M.E.H., Chakraborty B., Goldstein B.A., Ting D.S.W., Vaughan R., Liu N. (2022). Shapley variable importance cloud for interpretable machine learning. Patterns.

[42] Gomes R., Kamrowski C., Langlois J., Rozario P., Dircks I., Grottodden K., Martinez M., Tee W.Z., Sargeant K., LaFleur C. (2022). A Comprehensive Review of Machine Learning Used to Combat COVID-19. Diagnostics.

[43] Dogan O., Tiwari S., Jabbar M.A., Guggari S. (2021). A systematic review on AI/ML approaches against COVID-19 outbreak. Complex Intell. Syst..

[44] Gupta R.K., Marks M., Samuels T.H.A., Luintel A., Rampling T., Chowdhury H., Quartagno M., Nair A., Lipman M., Abubakar I. (2020). Systematic evaluation and external validation of 22 prognostic models among hospitalised adults with COVID-19: An observational cohort study. Eur. Respir. J..

[45] Khan M., Mehran M.T., Haq Z.U., Ullah Z., Naqvi S.R., Ihsan M., Abbass H. (2021). Applications of artificial intelligence in COVID-19 pandemic: A comprehensive review. Expert Syst. Appl..

[46] Syeda H.B., Syed M., Sexton K.W., Syed S., Begum S., Syed F., Prior F., Yu F. (2021). Role of Machine Learning Techniques to Tackle the COVID-19 Crisis: Systematic Review. JMIR Med. Inform..

[47] Kutikhin A.G., Shishkova D.K., Velikanova E.A., Sinitsky M.Y., Sinitskaya A.V., Markova V.E. (2022). Endothelial Dysfunction in the Context of Blood-Brain Barrier Modeling. J. Evol. Biochem. Physiol..

[48] Bogdanov L.A., Velikanova E.A., Kanonykina A.Y., Frolov A.V., Shishkova D.K., Lazebnaya A.I., Kutikhin A.G. (2022). Vascular smooth muscle cell contractile proteins as universal markers of vessels of microcirculatory bed. Compl. Iss. Cardiovasc. Dis..

[49] Shishkova D.K., Sinitskaya A.V., Sinitsky M.Y., Matveeva V.G., Velikanova E.A., Markova V.E., Kutikhin A.G. (2022). Spontaneous endothelial-to-mesenchymal transition in human primary umbilical vein endothelial cells. Compl. Iss. Cardiovasc. Dis..

[50] Vincent J.L., Levi M., Hunt B.J. (2022). Prevention and management of thrombosis in hospitalised patients with COVID-19 pneumonia. Lancet Respir. Med..

[51] Pelle M.C., Zaffina I., Lucà S., Forte V., Trapanese V., Melina M., Giofrè F., Arturi F. (2022). Endothelial Dysfunction in COVID-19: Potential Mechanisms and Possible Therapeutic Options. Life.

[52] Six I., Guillaume N., Jacob V., Mentaverri R., Kamel S., Boullier A., Slama M. (2022). The Endothelium and COVID-19: An Increasingly Clear Link Brief Title: Endotheliopathy in COVID-19. Int. J. Mol. Sci..

[53] Ambrosino P., Calcaterra I.L., Mosella M., Formisano R., D’Anna S.E., Bachetti T., Marcuccio G., Galloway B., Mancini F.P., Papa A. (2022). Endothelial Dysfunction in COVID-19: A Unifying Mechanism and a Potential Therapeutic Target. Biomedicines.

[54] Chee M.L., Ong M.E.H., Siddiqui F.J., Zhang Z., Lim S.L., Ho A.F.W., Liu N. (2021). Artificial Intelligence Applications for COVID-19 in Intensive Care and Emergency Settings: A Systematic Review. Int. J. Environ. Res. Public Health.

[55] Gao Y., Cai G.Y., Fang W., Li H.Y., Wang S.Y., Chen L., Yu Y., Liu D., Xu S., Cui P.F. (2020). Machine learning based early warning system enables accurate mortality risk prediction for COVID-19. Nat. Commun..

[56] Abdulaal A., Patel A., Charani E., Denny S., Alqahtani S.A., Davies G.W., Mughal N., Moore L.S.P. (2020). Comparison of deep learning with regression analysis in creating predictive models for SARS-CoV-2 outcomes. BMC Med. Inform. Decis. Mak..

[57] Abdulaal A., Patel A., Charani E., Denny S., Mughal N., Moore L. (2020). Prognostic Modeling of COVID-19 Using Artificial Intelligence in the United Kingdom: Model Development and Validation. J. Med. Internet Res..

[58] Italia L., Tomasoni D., Bisegna S., Pancaldi E., Stretti L., Adamo M., Metra M. (2021). COVID-19 and Heart Failure: From Epidemiology During the Pandemic to Myocardial Injury, Myocarditis, and Heart Failure Sequelae. Front. Cardiovasc. Med..

[59] Sokolski M., Reszka K., Suchocki T., Adamik B., Doroszko A., Drobnik J., Gorka-Dynysiewicz J., Jedrzejczyk M., Kaliszewski K., Kilis-Pstrusinska K. (2022). History of Heart Failure in Patients Hospitalized Due to COVID-19: Relevant Factor of In-Hospital Complications and All-Cause Mortality up to Six Months. J. Clin. Med..

[60] Cheng J., Sollee J., Hsieh C., Yue H., Vandal N., Shanahan J., Choi J.W., Tran T.M.L., Halsey K., Iheanacho F. (2022). COVID-19 mortality prediction in the intensive care unit with deep learning based on longitudinal chest X-rays and clinical data. Eur. Radiol..

[61] Kar S., Chawla R., Haranath S.P., Ramasubban S., Ramakrishnan N., Vaishya R., Sibal A., Reddy S. (2021). Multivariable mortality risk prediction using machine learning for COVID-19 patients at admission (AICOVID). Sci. Rep..

[62] Herzog A.L., von Jouanne-Diedrich H.K., Wanner C., Weismann D., Schlesinger T., Meybohm P., Stumpner J. (2021). COVID-19 and the kidney: A retrospective analysis of 37 critically ill patients using machine learning. PLoS ONE.

[63] Rechtman E., Curtin P., Navarro E., Nirenberg S., Horton M.K. (2020). Vital signs assessed in initial clinical encounters predict COVID-19 mortality in an NYC hospital system. Sci. Rep..

[64] Zhu J.S., Ge P., Jiang C., Zhang Y., Li X., Zhao Z., Zhang L., Duong T.Q. (2020). Deep-learning artificial intelligence analysis of clinical variables predicts mortality in COVID-19 patients. J. Am. Coll. Emerg. Physicians Open.

[65] Fu L., Wang B., Yuan T., Chen X., Ao Y., Fitzpatrick T., Li P., Zhou Y., Lin Y.F., Duan Q. (2020). Clinical characteristics of coronavirus disease 2019 (COVID-19) in China: A systematic review and meta-analysis. J. Infect..

[66] Lippi G., Plebani M. (2020). Laboratory abnormalities in patients with COVID-2019 infection. Clin. Chem. Lab. Med..

[67] Kim K.M., Evans D.S., Jacobson J., Jiang X., Browner W., Cummings S.R. (2022). Rapid prediction of in-hospital mortality among adults with COVID-19 disease. PLoS ONE.

[68] Lasso G., Khan S., Allen S.A., Mariano M., Florez C., Orner E.P., Quiroz J.A., Quevedo G., Massimi A., Hegde A. (2022). Longitudinally monitored immune biomarkers predict the timing of COVID-19 outcomes. PLoS Comput. Biol..

[69] Murri R., Lenkowicz J., Masciocchi C., Iacomini C., Fantoni M., Damiani A., Marchetti A., Sergi P.D.A., Arcuri G., Cesario A. (2021). A machine-learning parsimonious multivariable predictive model of mortality risk in patients with COVID-19. Sci. Rep..

[70] Snider J.M., You J.K., Wang X., Snider A.J., Hallmark B., Zec M.M., Seeds M.C., Sergeant S., Johnstone L., Wang Q. (2021). Group IIA secreted phospholipase A2 is associated with the pathobiology leading to COVID-19 mortality. J. Clin. Investig..

[71] Shiri I., Sorouri M., Geramifar P., Nazari M., Abdollahi M., Salimi Y., Khosravi B., Askari D., Aghaghazvini L., Hajianfar G. (2021). Machine learning-based prognostic modeling using clinical data and quantitative radiomic features from chest CT images in COVID-19 patients. Comput. Biol. Med..

