# Supplementary material for: Cardiovascular and Renal Comorbidities Included into Neural Networks Predict the Outcome in COVID-19 Patients Admitted to an Intensive Care Unit: Three-Center, Cross-Validation, Age- and Sex-Matched Study
Source: J Cardiovasc Dev Dis. 2023 Jan 23;10(2):39. doi: 10.3390/jcdd10020039 (PMC9967447; doi:10.3390/jcdd10020039)
Supplement: Supplementary file 1 [file jcdd-10-00039-s001.zip › jcdd-2095172-supplementary.pdf]

Table S1. Optimal parameters for the best models with regards to each machine learning approach.

Model	Parameters	Best Value
Decision trees (sklearn.tree.DecisionTreeClassifier)	'ccp_alpha'	0
	'class_weight'	None
	'criterion'	'entropy'
	'max_depth'	4
	'max_features'	None
	'max_leaf_nodes'	None
	'min_impurity_decrease'	0
	'min_samples_leaf'	1
	'min_samples_split'	2
	'min_weight_fraction_leaf'	0
	'random_state'	1236
	'splitter'	'best'
Random forests (sklearn.ensemble.RandomForestClassifier)	'bootstrap'	True
	'ccp_alpha'	0
	'class_weight'	None
	'criterion'	'gini'
	'max_depth'	6
	'max_features'	0.8
	'max_leaf_nodes'	None
	'max_samples'	None
	'min_impurity_decrease'	0
	'min_samples_leaf'	1
	'min_samples_split'	30
	'min_weight_fraction_leaf'	0
	'n_estimators'	200
	'n_jobs'	-1
	'oob_score'	False
	'random_state'	1236
	'verbose'	0
	'warm_start'	True
Extra trees (sklearn.ensemble.ExtraTreesClassifier)	'bootstrap'	False
	'ccp_alpha'	0
	'class_weight'	None
	'criterion'	'entropy'
	'max_depth'	7
	'max_features'	0.6
	'max_leaf_nodes'	None
	'max_samples'	None
	'min_impurity_decrease'	0
	'min_samples_leaf'	1
	'min_samples_split'	50
	'min_weight_fraction_leaf'	0
	'n_estimators'	200
	'n_jobs'	-1
	'oob_score'	False

	'random_state'	1238
	'verbose'	0
	'warm_start'	True
Neural networks (sklearn.neural_network.MLPClassifier)	'activation'	'relu'
	'alpha'	0.0001
	'batch_size'	'auto'
	'beta_1'	0.9
	'beta_2'	0.999
	'early_stopping'	True
	'epsilon'	1.00E-08
	'hidden_layer_sizes'	(16, 32)
	'learning_rate'	'constant'
	'learning_rate_init'	0.1
	'max_fun'	15000
	'max_iter'	500
	'momentum'	0.9
	'n_iter_no_change'	50
	'nesterovs_momentum'	True
	'power_t'	0.5
	'random_state'	1243
	'shuffle'	True
	'solver'	'adam'
	'tol'	0.0001
	'validation_fraction'	0.1
	'verbose'	False
	'warm_start'	False
K-nearest neighbours (sklearn.neighbors.KNeighborsClassifier)	'algorithm'	'kd_tree'
	'leaf_size'	30
	'metric'	'minkowski'
	'metric_params'	None
	'n_jobs'	-1
	'n_neighbors'	7
	'p'	2
	'weights'	'distance'
XGBoost (XGBoost.XGBClassifier)	'objective'	'binary logistic'
	'base_score'	0.5
	'booster'	'gbtree'
	'colsample_bylevel'	1
	'colsample_bynode'	1
	'colsample_bytree'	1
	'eval_metric'	None
	'gamma'	0
	'gpu_id'	-1
	'grow_policy'	'depthwise'
	'interaction_constraints'	''
	'learning_rate'	0.300000012
	'max_bin'	256
	'max_cat_to_onehot'	4

	'max_delta_step'	0
	'max_depth'	6
	'max_leaves'	0
	'min_child_weight'	1
	'monotone_constraints'	'()'
	'n_jobs'	0
	'num_parallel_tree'	1
	'predictor'	'auto'
	'random_state'	0
	'reg_alpha'	0
	'reg_lambda'	1
	'sampling_method'	'uniform'
	'scale_pos_weight'	1
	'subsample'	1
	'tree_method'	'auto'
	'validate_parameters'	1
	'verbosity'	None
LightGBM (lightgbm.LGBMClassifier)	'bagging_fraction'	0.5
	'bagging_freq'	0
	'bagging_seed'	31935
	'boosting'	'gbdt'
	'cat_smooth'	10
	'cegb_penalty_split'	0
	'cegb_tradeoff'	1
	'data_random_seed'	4087
	'deterministic'	0
	'device_type'	'cpu'
	'drop_rate'	0.1
	'early_stopping_round'	0
	'extra_seed'	31278
	'extra_trees'	0
	'feature_fraction'	0.5
	'feature_fraction_bynode'	1
	'feature_fraction_seed'	22066
	'first_metric_only'	0
	'force_col_wise'	0
	'force_row_wise'	0
	'histogram_pool_size'	-1
	'lambda_l1'	0
	'lambda_l2'	0
	'learning_rate'	0.05
	'linear_lambda'	0
	'max_cat_threshold'	32
	'max_cat_to_onehot'	4
	'max_delta_step'	0
	'max_depth'	-1
	'max_drop'	50
	'min_data_in_leaf'	15

	'min_data_per_group'	100
	'min_sum_hessian_in_leaf'	0.001
	'monotone_constraints_method'	'basic'
	'monotone_penalty'	0
	'neg_bagging_fraction'	1
	'num_iterations'	10000
	'num_leaves'	127
	'num_threads'	0
	'objective'	'binary'
	'other_rate'	0.1
	'path_smooth'	0
	'pos_bagging_fraction'	1
	'refit_decay_rate'	0.9
	'saved_feature_importance_type'	0
	'skip_drop'	0.5
	'top_k'	20
	'top_rate'	0.2
	'tree_learner'	'serial'
	'verbosity'	-1
CatBoost (catboost.CatBoostClassifier)	'nan_mode'	'Min'
	'eval_metric'	'AUC'
	'iterations'	1000
	'sampling_frequency'	'PerTree'
	'leaf_estimation_method'	'Newton'
	'od_pval'	0
	'grow_policy'	'SymmetricTree'
	'penalties_coefficient'	1
	'boosting_type'	'Plain'
	'model_shrink_mode'	'Constant'
	'feature_border_type'	'GreedyLogSum'
	'bayesian_matrix_reg'	0.100000002
	'eval_fraction'	0
	'force_unit_auto_pair_weights'	False
	'l2_leaf_reg'	1.863051295
	'random_strength'	5.292492867
	'od_type'	'Iter'
	'rsm'	0.116269194
	'boost_from_average'	False
	'model_size_reg'	0.5
	'pool_metainfo_options'	{'tags' {}}
	'subsample'	0.800000012
	'use_best_model'	True
	'od_wait'	50
	'class_names'	[0,1]
	'random_seed'	1234
	'depth'	3
	'posterior_sampling'	False
	'border_count'	254

	'classes_count'	0
	'auto_class_weights'	'None'
	'sparse_features_conflict_fraction'	0
	'leaf_estimation_backtracking'	'AnyImprovement'
	'best_model_min_trees'	1
	'model_shrink_rate'	0
	'min_data_in_leaf'	65
	'loss_function'	'Logloss'
	'learning_rate'	0.100000002
	'score_function'	'Cosine'
	'task_type'	'CPU'
	'leaf_estimation_iterations'	10
	'bootstrap_type'	'MVS'
	'max_leaves'	8
Multivariate logistic regression (sklearn.linear_model.LogisticRegression)	'C'	1
	'class_weight'	None
	'dual'	False
	'fit_intercept'	True
	'intercept_scaling'	1
	'l1_ratio'	None
	'max_iter'	500
	'multi_class'	'auto'
	'n_jobs'	-1
	'penalty'	'l2'
	'random_state'	None
	'solver'	'lbfgs'
	'tol'	0.0005
	'verbose'	0
	'warm_start'	False

Table S2. Correlation matrix of continuous predictors of COVID-19-related death in patients who has been admitted to the intensive care unit (ICU) because of severe COVID-19.

**Parameters**	Age, years	WBC, × 10^9/L	NE#, × 10^9/L	LY#, × 10^9/L	NLR	PLT, × 10^9/L	BUN, mmol/L	sCr, µmol/L	GFR, mL/min/ /1.73 m^2	AST, U/L	ALT, U/L	FPG, mmol/L	CRP, mg/L	D-dimer, ng/mL
Age, years	1.00	−0.08	−0.11	−0.03	−0.03	−0.10	0.18	0.23	**−0.44**	−0.22	−0.27	0.07	−0.09	−0.07
WBC, × 10^9/L	−0.08	1.00	**0.92**	0.13	**0.51**	0.28	**0.31**	0.09	−0.01	0.15	0.12	0.08	0.12	0.23
NE#, × 10^9/L	−0.11	**0.92**	1.00	−0.03	**0.67**	0.24	**0.32**	0.11	−0.02	0.18	0.14	0.12	0.19	0.25
LY#, × 10^9/L	−0.03	0.13	−0.03	1.00	**−0.72**	0.16	−0.03	−0.04	0.06	0.00	0.04	−0.20	−0.22	−0.06
NLR	−0.03	**0.51**	**0.67**	**−0.72**	1.00	0.02	0.21	0.09	−0.06	0.11	0.05	0.20	0.27	0.17
PLT, × 10^9/L	−0.10	0.28	0.24	0.16	0.02	1.00	−0.07	−0.14	0.11	−0.02	0.07	−0.04	0.07	0.08
BUN, mmol/L	0.18	**0.31**	**0.32**	−0.03	0.21	−0.07	1.00	**0.62**	**−0.54**	0.06	0.04	0.16	−0.01	0.09
sCr, µmol/L	0.23	0.09	0.11	−0.04	0.09	−0.14	**0.62**	1.00	**−0.89**	0.02	−0.11	0.10	−0.07	−0.14
GFR, mL/min/1.73 m^2	**−0.44**	−0.01	−0.02	0.06	−0.06	0.11	**−0.54**	**−0.89**	1.00	0.06	0.20	−0.14	0.05	0.16
AST, U/L	−0.22	0.15	0.18	0.00	0.11	−0.02	0.06	0.02	0.06	1.00	**0.66**	0.09	0.07	−0.05
ALT, U/L	−0.27	0.12	0.14	0.04	0.05	0.07	0.04	−0.11	0.20	**0.66**	1.00	0.09	−0.07	0.00
FPG, mmol/L	0.07	0.08	0.12	−0.20	0.20	−0.04	0.16	0.10	−0.14	0.09	0.09	1.00	0.06	0.01
CRP, mg/L	−0.09	0.12	0.19	−0.22	0.27	0.07	−0.01	−0.07	0.05	0.07	−0.07	0.06	1.00	0.26
D-dimer, ng/mL	−0.07	0.23	0.25	−0.06	0.17	0.08	0.09	−0.14	0.16	−0.05	0.00	0.01	0.26	1.00

Table S3. Classification summary and AUROC of neural networks for the prediction of fatal outcome in patients admitted to the ICU with severe COVID-19 (merged dataset from three centers). Manually selected predictors (CAD/CHF, stage 3-5 CKD, BUN, and CRP).

Cross-validation sample						Average bootstrapping					
**Neural network**	**n, %**	**Hospital discharge**	**In-hospital death**	**All**	**AUROC**	**Neural network**	**n, %**	**Hospital discharge**	**In-hospital death**	**All**	**AUROC**
1	Total (n)	57	47	104	0.850	1	Total (n)	52.25	51.75	104	0.823
	Correct (n)	47	38	85			Correct (n)	41.5	39	80.5	
	Incorrect (n)	10	9	19			Incorrect (n)	10.75	12.75	23.5	
	Correct (%)	82.46	80.85	81.73			Correct (%)	79.36	75.19	77.40	
	Incorrect (%)	17.54	19.15	18.27			Incorrect (%)	20.64	24.81	22.60	
2	Total (n)	57	47	104	0.834	2	Total (n)	52.25	51.75	104	0.804
	Correct (n)	46	38	84			Correct (n)	40	39	79	
	Incorrect (n)	11	9	20			Incorrect (n)	12.25	12.75	25	
	Correct (%)	80.70	80.85	80.77			Correct (%)	76.49	75.19	75.96	
	Incorrect (%)	19.30	19.15	19.23			Incorrect (%)	23.51	24.81	24.04	
3	Total (n)	57	47	104	0.834	3	Total (n)	52.25	51.75	104	0.809
	Correct (n)	42	43	85			Correct (n)	37.25	47	84.25	
	Incorrect (n)	15	4	19			Incorrect (n)	15	4.75	19.75	
	Correct (%)	73.68	91.49	81.73			Correct (%)	71.45	90.55	81.01	
	Incorrect (%)	26.32	8.51	18.27			Incorrect (%)	28.55	9.45	18.99	
4	Total (n)	57	47	104	0.846	4	Total (n)	52.25	51.75	104	0.820
	Correct (n)	42	43	85			Correct (n)	37.25	47	84.25	
	Incorrect (n)	15	4	19			Incorrect (n)	15	4.75	19.75	
	Correct (%)	73.68	91.49	81.73			Correct (%)	71.45	90.55	81.01	
	Incorrect (%)	26.32	8.51	18.27			Incorrect (%)	28.55	9.45	18.99	
5	Total (n)	57	47	104	0.849	5	Total (n)	52.25	51.75	104	0.821
	Correct (n)	42	43	85			Correct (n)	37.25	47	84.25	
	Incorrect (n)	15	4	19			Incorrect (n)	15	4.75	19.75	
	Correct (%)	73.68	91.49	81.73			Correct (%)	71.45	90.55	81.01	

	Incorrect (%)	26.32	8.51	18.27			Incorrect (%)	28.55	9.45	18.99	
6	Total (n)	57	47	104	0.823	6	Total (n)	52.25	51.75	104	0.799
	Correct (n)	42	43	85			Correct (n)	37.25	47	84.25	
	Incorrect (n)	15	4	19			Incorrect (n)	15	4.75	19.75	
	Correct (%)	73.68	91.49	81.73			Correct (%)	71.45	90.55	81.01	
	Incorrect (%)	26.32	8.51	18.27			Incorrect (%)	28.55	9.45	18.99	
7	Total (n)	57	47	104	0.837	7	Total (n)	52.25	51.75	104	0.812
	Correct (n)	42	43	85			Correct (n)	37.25	47	84.25	
	Incorrect (n)	15	4	19			Incorrect (n)	15	4.75	19.75	
	Correct (%)	73.68	91.49	81.73			Correct (%)	71.45	90.55	81.01	
	Incorrect (%)	26.32	8.51	18.27			Incorrect (%)	28.55	9.45	18.99	
8	Total (n)	57	47	104	0.845	8	Total (n)	52.25	51.75	104	0.849
	Correct (n)	44	41	85			Correct (n)	37.25	47	84.25	
	Incorrect (n)	13	6	19			Incorrect (n)	15	4.75	19.75	
	Correct (%)	77.19	87.23	81.73			Correct (%)	71.45	90.55	81.01	
	Incorrect (%)	22.81	12.77	18.27			Incorrect (%)	28.55	9.45	18.99	
9	Total (n)	57	47	104	0.861	9	Total (n)	52.25	51.75	104	0.832
	Correct (n)	42	43	85			Correct (n)	39.25	43.75	83	
	Incorrect (n)	15	4	19			Incorrect (n)	13	8	21	
	Correct (%)	73.68	91.49	81.73			Correct (%)	75.12	84.32	79.81	
	Incorrect (%)	26.32	8.51	18.27			Incorrect (%)	24.88	15.68	20.19	
10	Total (n)	57	47	104	0.866	10	Total (n)	52.25	51.75	104	0.849
	Correct (n)	44	41	85			Correct (n)	39.25	43.75	83	
	Incorrect (n)	13	6	19			Incorrect (n)	13	8	21	
	Correct (%)	77.19	87.23	81.73			Correct (%)	75.12	84.32	79.81	
	Incorrect (%)	22.81	12.77	18.27			Incorrect (%)	24.88	15.68	20.19	
11	Total (n)	57	47	104	0.837	11	Total (n)	52.25	51.75	104	0.814

	Correct (n)	42	43	85			Correct (n)	37.25	47	84.25	
	Incorrect (n)	15	4	19			Incorrect (n)	15	4.75	19.75	
	Correct (%)	73.68	91.49	81.73			Correct (%)	71.45	90.55	81.01	
	Incorrect (%)	26.32	8.51	18.27			Incorrect (%)	28.55	9.45	18.99	
12	Total (n)	57	47	104	0.853	12	Total (n)	52.25	51.75	104	0.835
	Correct (n)	42	43	85			Correct (n)	37.25	47	84.25	
	Incorrect (n)	15	4	19			Incorrect (n)	15	4.75	19.75	
	Correct (%)	73.68	91.49	81.73			Correct (%)	71.45	90.55	81.01	
	Incorrect (%)	26.32	8.51	18.27			Incorrect (%)	28.55	9.45	18.99	
13	Total (n)	57	47	104	0.842	13	Total (n)	52.25	51.75	104	0.819
	Correct (n)	42	43	85			Correct (n)	37.25	47	84.25	
	Incorrect (n)	15	4	19			Incorrect (n)	15	4.75	19.75	
	Correct (%)	73.68	91.49	81.73			Correct (%)	71.45	90.55	81.01	
	Incorrect (%)	26.32	8.51	18.27			Incorrect (%)	28.55	9.45	18.99	
14	Total (n)	57	47	104	0.839	14	Total (n)	52.25	51.75	104	0.809
	Correct (n)	46	38	84			Correct (n)	40	39	79	
	Incorrect (n)	11	9	20			Incorrect (n)	12.25	12.75	25	
	Correct (%)	80.70	80.85	80.77			Correct (%)	76.63	75.19	75.96	
	Incorrect (%)	19.30	19.15	19.23			Incorrect (%)	23.37	24.81	24.04	
15	Total (n)	57	47	104	0.825	15	Total (n)	52.25	51.75	104	0.808
	Correct (n)	42	43	85			Correct (n)	37.25	47	84.25	
	Incorrect (n)	15	4	19			Incorrect (n)	15	4.75	19.75	
	Correct (%)	73.68	91.49	81.73			Correct (%)	71.45	90.55	81.01	
	Incorrect (%)	26.32	8.51	18.27			Incorrect (%)	28.55	9.45	18.99	

Table S4. Classification summary and AUROC of neural networks for the prediction of fatal outcome in patients admitted to the ICU with severe COVID-19 (merged dataset from three centers). Predictors identified by Predictor Screening tool of STATISTICA software (CAD/CHF, LY#, NLR, and CRP).

	Cross-validation sample						Average bootstrapping				
**Neural network**	**n, %**	**Hospital discharge**	**In-hospital death**	**All**	**AUROC**	**Neural network**	**n, %**	**Hospital discharge**	**In-hospital death**	**All**	**AUROC**
1	Total (n)	57	47	104	0.820	1	Total (n)	52	52	104	0.775
	Correct (n)	46	38	84			Correct (n)	41	39	79	
	Incorrect (n)	11	9	20			Incorrect (n)	12	13	25	
	Correct (%)	80.70	80.85	80.77			Correct (%)	77.47	74.85	76.20	
	Incorrect (%)	19.30	19.15	19.23			Incorrect (%)	22.53	25.15	23.80	
2	Total (n)	57	47	104	0.824	2	Total (n)	52	52	104	0.778
	Correct (n)	47	37	84			Correct (n)	42	38	79	
	Incorrect (n)	10	10	20			Incorrect (n)	11	14	25	
	Correct (%)	82.46	78.72	80.77			Correct (%)	79.38	72.35	75.96	
	Incorrect (%)	17.54	21.28	19.23			Incorrect (%)	20.62	27.65	24.04	
3	Total (n)	57	47	104	0.815	3	Total (n)	52	52	104	0.770
	Correct (n)	46	38	84			Correct (n)	40	39	78	
	Incorrect (n)	11	9	20			Incorrect (n)	13	13	26	
	Correct (%)	80.70	80.85	80.77			Correct (%)	75.57	74.85	75.24	
	Incorrect (%)	19.30	19.15	19.23			Incorrect (%)	24.43	25.15	24.76	
4	Total (n)	57	47	104	0.817	4	Total (n)	52	52	104	0.772
	Correct (n)	46	38	84			Correct (n)	40	39	78	
	Incorrect (n)	11	9	20			Incorrect (n)	13	13	26	
	Correct (%)	80.70	80.85	80.77			Correct (%)	75.57	74.85	75.24	
	Incorrect (%)	19.30	19.15	19.23			Incorrect (%)	24.43	25.15	24.76	
5	Total (n)	57	47	104	0.815	5	Total (n)	52	52	104	0.779
	Correct (n)	46	39	85			Correct (n)	42	40	82	
	Incorrect (n)	11	8	19			Incorrect (n)	10	12	22	
	Correct (%)	80.70	82.98	81.73			Correct (%)	80.50	77.26	78.85	
	Incorrect (%)	19.30	17.02	18.27			Incorrect (%)	19.50	22.74	21.15	
6	Total (n)	57	47	104	0.819	6	Total (n)	52	52	104	0.773
	Correct (n)	46	38	84			Correct (n)	40	39	78	
	Incorrect (n)	11	9	20			Incorrect (n)	13	13	26	
	Correct (%)	80.70	80.85	80.77			Correct (%)	75.57	74.85	75.24	
	Incorrect (%)	19.30	19.15	19.23			Incorrect (%)	24.43	25.15	24.76	
7	Total (n)	57	47	104	0.818	7	Total (n)	52	52	104	0.773

	Correct (n)	46	38	84			Correct (n)	40	39	78	
	Incorrect (n)	11	9	20			Incorrect (n)	13	13	26	
	Correct (%)	80.70	80.85	80.77			Correct (%)	75.57	74.85	75.24	
	Incorrect (%)	19.30	19.15	19.23			Incorrect (%)	24.43	25.15	24.76	
	Total (n)	57	47	104			Total (n)	52	52	104	
	Correct (n)	47	38	85			Correct (n)	41	39	80	
8	Incorrect (n)	10	9	19	0.820	8	Incorrect (n)	11	13	24	0.775
	Correct (%)	82.46	80.85	81.73			Correct (%)	78.98	74.85	76.92	
	Incorrect (%)	17.54	19.15	18.27			Incorrect (%)	21.02	25.15	23.08	
	Total (n)	57	47	104			Total (n)	52	52	104	
	Correct (n)	47	38	85			Correct (n)	41	39	80	
9	Incorrect (n)	10	9	19	0.817	9	Incorrect (n)	11	13	24	0.769
	Correct (%)	82.46	80.85	81.73			Correct (%)	78.34	74.85	76.68	
	Incorrect (%)	17.54	19.15	18.27			Incorrect (%)	21.66	25.15	23.32	
	Total (n)	57	47	104			Total (n)	52	52	104	
	Correct (n)	47	37	84			Correct (n)	41	38	79	
10	Incorrect (n)	10	10	20	0.817	10	Incorrect (n)	11	14	25	0.772
	Correct (%)	82.46	78.72	80.77			Correct (%)	78.98	73.35	76.20	
	Incorrect (%)	17.54	21.28	19.23			Incorrect (%)	21.02	26.65	23.80	

Table S5. Classification summary and AUROC of neural networks for the prediction of fatal outcome in patients admitted to the ICU with severe COVID-19 (merged dataset from three centers). Predictors combined from the manual screening and Predictor Screening tool of STATISTICA software (CAD/CHF, stage 3-5 CKD, BUN, LY#, NLR, and CRP).

Cross-validation sample					
**Neural network**	**n, %**	**Hospital discharge**	**In-hospital death**	**All**	**AUROC**
1	Total (n)	57	47	104	0.838
	Correct (n)	46	39	85	
	Incorrect (n)	11	8	19	
	Correct (%)	80.70	82.98	81.73	
	Incorrect (%)	19.30	17.02	18.27	
2	Total (n)	57	47	104	0.832
	Correct (n)	47	38	85	
	Incorrect (n)	10	9	19	
	Correct (%)	82.46	80.85	81.73	
	Incorrect (%)	17.54	19.15	18.27	
3	Total (n)	57	47	104	0.836
	Correct (n)	47	38	85	
	Incorrect (n)	10	9	19	
	Correct (%)	82.46	80.85	81.73	
	Incorrect (%)	17.54	19.15	18.27	
4	Total (n)	57	47	104	0.831
	Correct (n)	47	38	85	
	Incorrect (n)	10	9	19	
	Correct (%)	82.46	80.85	81.73	
	Incorrect (%)	17.54	19.15	18.27	
5	Total (n)	57	47	104	0.824
	Correct (n)	46	39	85	
	Incorrect (n)	11	8	19	
	Correct (%)	80.70	82.98	81.73	
	Incorrect (%)	19.30	17.02	18.27	
6	Total (n)	57	47	104	0.835
	Correct (n)	47	38	85	
	Incorrect (n)	10	9	19	
	Correct (%)	82.46	80.85	81.73	
	Incorrect (%)	17.54	19.15	18.27	
7	Total (n)	57	47	104	0.825
	Correct (n)	47	38	85	
	Incorrect (n)	10	9	19	
	Correct (%)	82.46	80.85	81.73	
	Incorrect (%)	17.54	19.15	18.27	
8	Total (n)	57	47	104	0.831
	Correct (n)	47	38	85	
	Incorrect (n)	10	9	19	
	Correct (%)	82.46	80.85	81.73	
	Incorrect (%)	17.54	19.15	18.27	
9	Total (n)	57	47	104	0.833
	Correct (n)	47	38	85	
	Incorrect (n)	10	9	19	
	Correct (%)	82.46	80.85	81.73	

	Incorrect (%)	17.54	19.15	18.27	
10	Total (n)	57	47	104	0.832
	Correct (n)	47	38	85	
	Incorrect (n)	10	9	19	
	Correct (%)	82.46	80.85	81.73	
	Incorrect (%)	17.54	19.15	18.27	
11	Total (n)	57	47	104	0.830
	Correct (n)	47	38	85	
	Incorrect (n)	10	9	19	
	Correct (%)	82.46	80.85	81.73	
	Incorrect (%)	17.54	19.15	18.27	
12	Total (n)	57	47	104	0.827
	Correct (n)	47	38	85	
	Incorrect (n)	10	9	19	
	Correct (%)	82.46	80.85	81.73	
	Incorrect (%)	17.54	19.15	18.27	
13	Total (n)	57	47	104	0.832
	Correct (n)	47	38	85	
	Incorrect (n)	10	9	19	
	Correct (%)	82.46	80.85	81.73	
	Incorrect (%)	17.54	19.15	18.27	
14	Total (n)	57	47	104	0.835
	Correct (n)	46	39	85	
	Incorrect (n)	11	8	19	
	Correct (%)	80.70	82.98	81.73	
	Incorrect (%)	19.30	17.02	18.27	
15	Total (n)	57	47	104	0.825
	Correct (n)	47	38	85	
	Incorrect (n)	10	9	19	
	Correct (%)	82.46	80.85	81.73	
	Incorrect (%)	17.54	19.15	18.27	
